# Undifferentiated Pleomorphic Sarcoma and Hyperparathyroidism in an Adolescent Male: A Case Report and Review of Hyperparathyroidism-associated Sarcomas

**DOI:** 10.5435/JAAOSGlobal-D-19-00125

**Published:** 2020-02-10

**Authors:** Stephen A. Herrmann, Rupert Stanborough, John S. A. Chrisinger, Jack W. Jennings

**Affiliations:** From the Mallinckrodt Institute of Radiology (Herrmann, Stanborough, Jennings), and the Department of Pathology and Immunology (Chrisinger), Washington University, St. Louis, MO.

## Abstract

The association between hyperparathyroidism and sarcoma is extremely rare with other reported cases describing the development of osteosarcoma and chondrosarcomas in middle-aged adults. This case describes an adolescent male with hyperparathyroidism and a pathologic fracture of a biopsy-proven brown tumor in the distal right femur. The fracture healed but later developed an undifferentiated pleomorphic sarcoma of the bone at the site of the known brown tumor. Although in vitro and in vivo studies have demonstrated the risks of elevated parathyroid hormone with development of sarcomas, there is limited evidence of a human association. The effects of elevated parathyroid hormone on the skeletally immature bone in the setting of sarcoma formation are currently not well understood without current description of adolescent hyperparathyroidism-associated sarcomas. This case highlights a sarcoma originating at a pathologically proven brown tumor within an adolescent male, discusses the association of sarcoma with hyperparathyroidism, and reviews the other nine reported cases in the literature.

Development of bone sarcomas in patients with hyperparathyroidism is rare, with nine other reported cases in the literature to our knowledge.^1,2,3,4,5,6^ Parathyroid hormone (PTH) is a primary modulator of bone remodeling in the setting of calcium homeostasis. PTH effects on modulating osteoblasts and gene expression has been shown to alter skeletal structure,^7-9^ and PTH analogs have been used subsequently for treatment of osteoporosis.^10^ It has also been suggested that PTH may stimulate abnormal osteoblastic activity, and an association between elevated PTH and risk for osteosarcoma formation has been demonstrated in several in vitro^11,12^ and rat studies.^13^ Based on these findings in rat models, there have been several human cohort studies^6,14,15^ evaluating whether there is a relationship between PTH and sarcomas. We present a unique case of a young man with a history of adolescent primary hyperparathyroidism and fracture through a histologically proven brown tumor with subsequent development of an undifferentiated pleomorphic sarcoma (UPS) of the bone to make clinicians aware of this rare presentation. Identifying a possible association between sarcoma development in hyperparathyroidism is important because the delayed diagnosis of both can lead to poor clinical outcomes.

## Case Report

**Figure 1:**
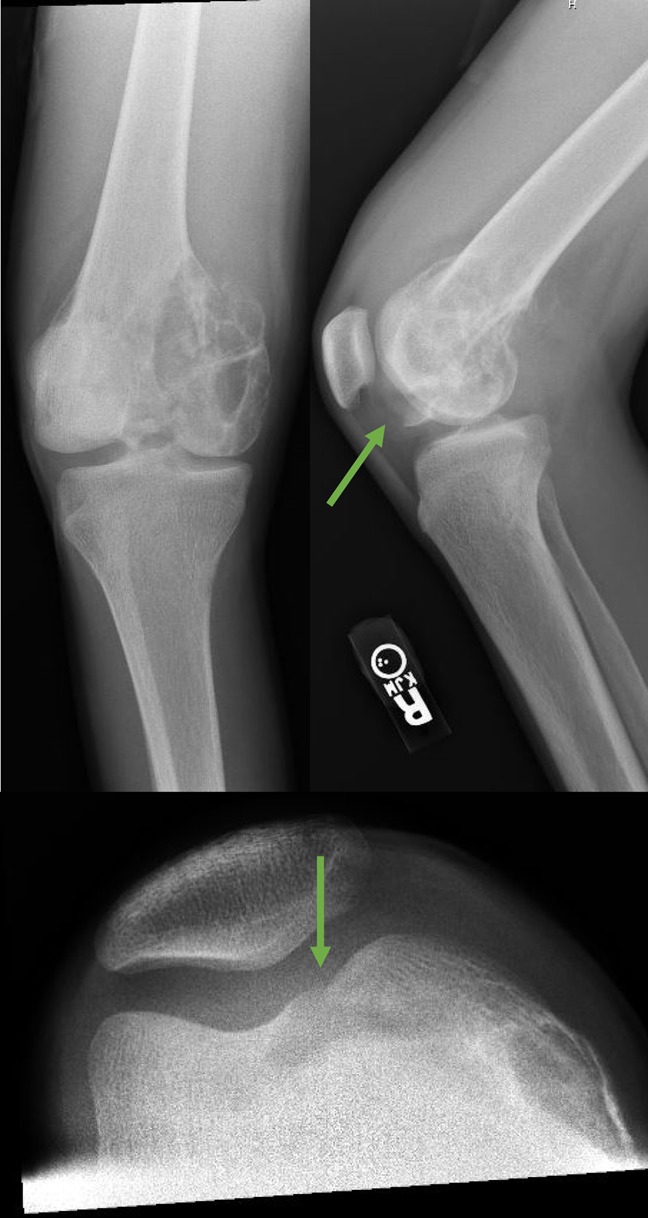
A radiograph 3 years before presentation demonstrating a lytic lesion within the right knee medial femoral condyle with associated pathologic fracture (green arrows), which was histologically proven to be a brown tumor.

**Figure 2:**
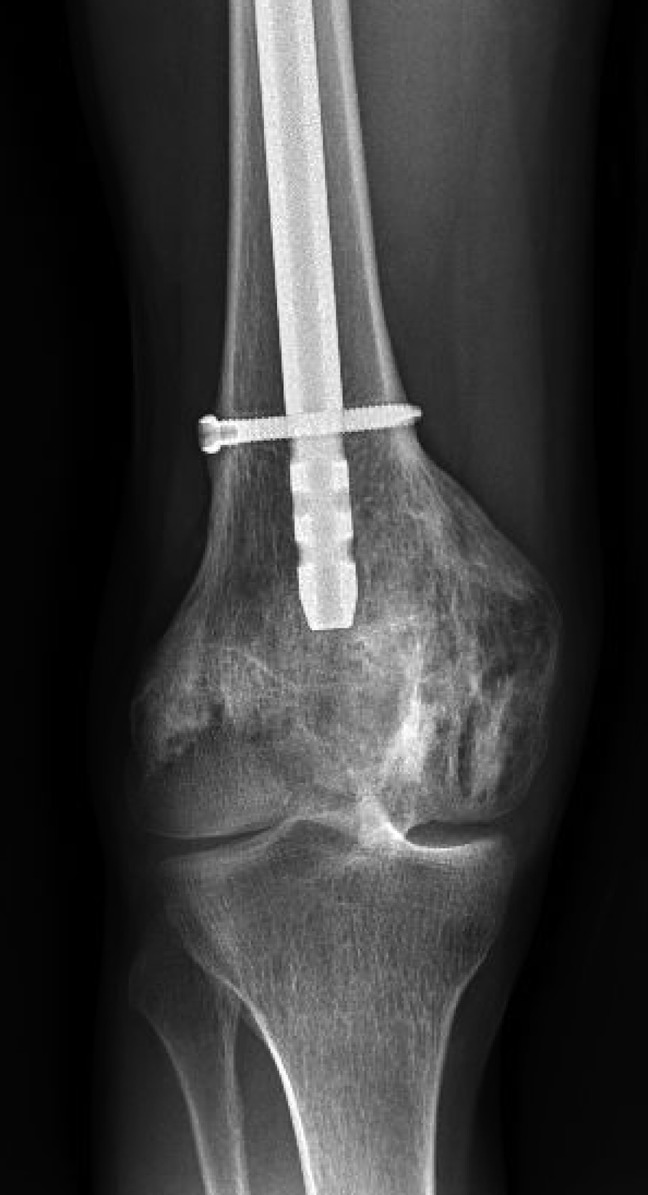
A radiograph 2 years before presentation showing a nailed right mid femoral shaft fracture and healed medial femoral condyle fracture with increased sclerosis of the brown tumor.

**Figure 3:**
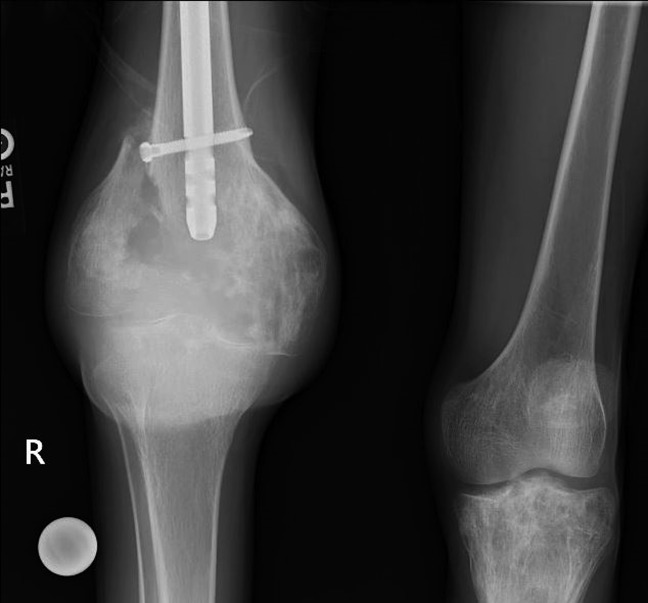
A radiograph 1 month before presentation demonstrating a pathologic fracture of the distal right femur at the apex of the intramedullary nail and extending to the biopsy-proven brown tumor. Left proximal tibia lesion with features of an additional brown tumor.

**Figure 4:**
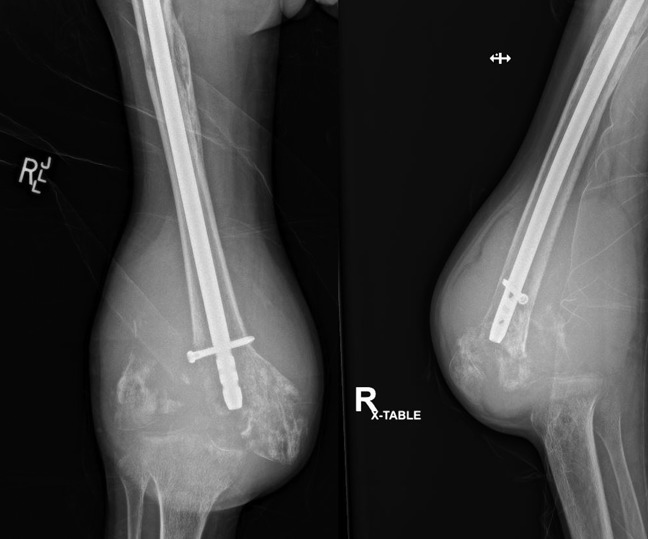
A radiograph at our initial clinical encounter demonstrating increased fracture displacement and osseous destruction of the distal right femur fracture with soft-tissue component.

**Figure 5:**
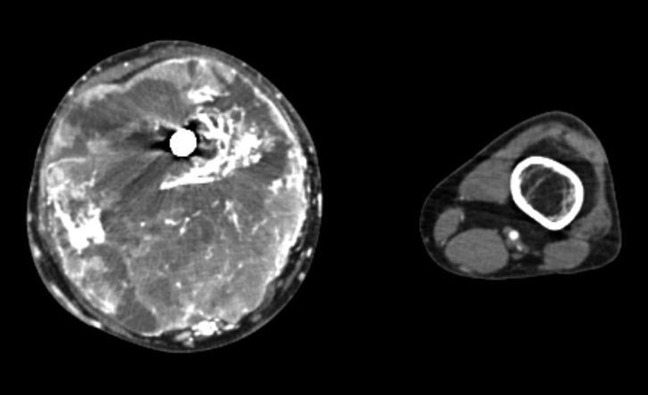
Photograph showing CT contrast-enhanced transaxial imaging demonstrating a large soft-tissue lesion with necrosis and osseous destruction of the distal femur.

**Figure 6:**
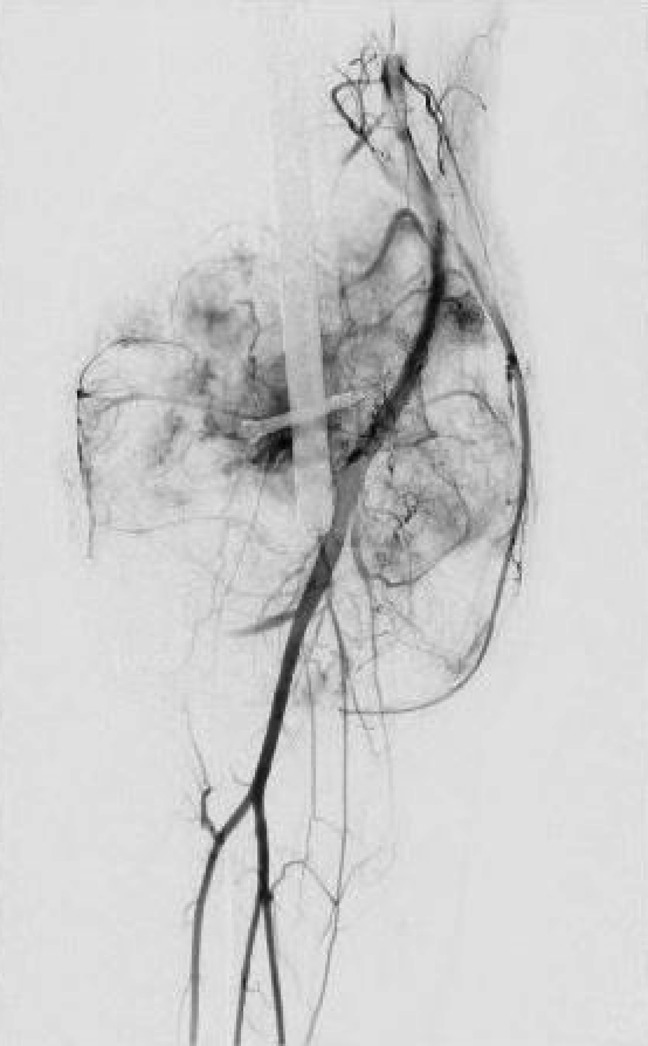
Angiogram of the right lower extremity showing patency of the right popliteus artery and hypervascularity of the soft-tissue lesion.

**Figure 7:**
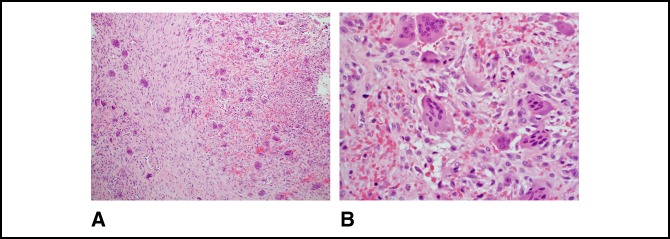
**A**, Micrograph showing a tumor composed of a haphazard and fascicular proliferation of bland spindle cells and osteoclast-type giant cells. Giant cells have an uneven distribution and cluster around the areas of hemorrhage (100×, H&E). **B**, Micrograph showing spindle cells with a disorderly arrangement with extravasated red blood cells and giant cells. The nuclear features of the spindle cells and giant cells are distinct from one another and significant atypia is absent (400×, H&E).

**Figure 8:**
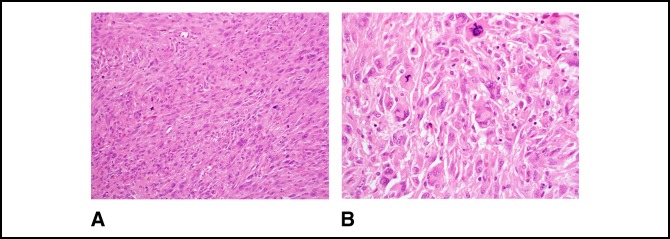
**A**, Photograph showing microscopically markedly atypical spindle cells arranged in fascicles with brisk mitotic activity including atypical forms. Scattered tumor giant cells and epithelioid cells are also present (200×, H&E). **B**, Photograph showing other areas show more sheet-like growth with elongated and polygonal cells. Tumor giant cells and atypical mitotic figures are conspicuous (400×, H&E).

**Figure 9:**
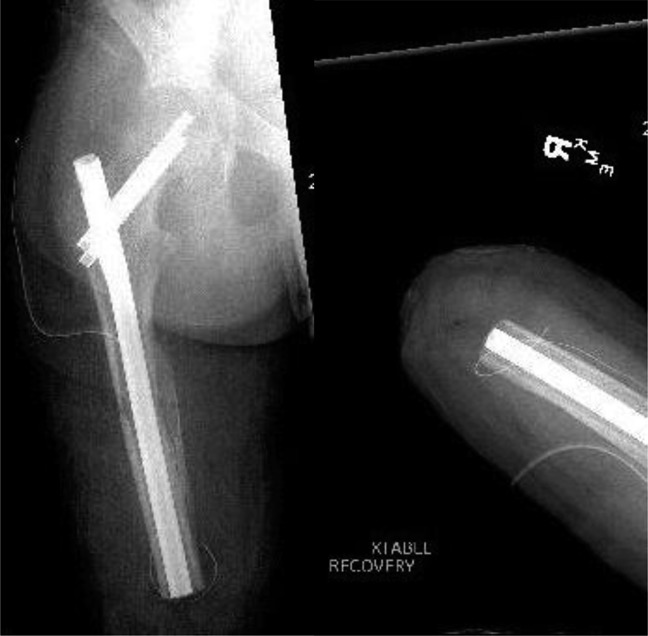
Radiographs of the right femur after above-the-knee amputation.

A 22-year-old man presented to our clinic for right distal femur pathologic fracture management in the setting of treated primary hyperparathyroidism. Three years before presentation, he had a right femur medial femoral condyle pathologic fracture through a lytic lesion, which was histologically diagnosed as a brown tumor (Figure 1) at an outside facility and primary hyperparathyroidism with an elevated serum calcium (16.8 units) and PTH (389 units). A bone scan revealed multiple lesions within the pelvis, skull, femora, and tibiae which were favored to represent additional brown tumors. He was managed by an endocrinologist with eventual parathyroid adenoma resection with resultant resolution of hyperparathyroidism. Two years before presentation, he was in a motor vehicle accident and fractured his right mid femoral shaft, managed with intramedullary nailing. Follow-up radiographs confirmed the distal femur pathologic fracture healed with progressive brown tumor sclerosis (Figure 2). Two months before presentation, he had a distal right femur periprosthetic fracture involving the medial femoral condyle brown tumor (Figure 3). At the time of current presentation, his hyperparathyroidism was well controlled with normal calcium and PTH levels. While awaiting surgical clearance, he had progressive right knee swelling; radiographs obtained after our initial clinic visit demonstrated destructive changes of the distal femur fracture and associated soft-tissue mass (Figure 4). CT imaging and angiography confirmed osseous destruction and soft-tissue mass displacing popliteus vasculature (Figures 5 and 6). Biopsy and histology of the lesion demonstrated a high-grade sarcoma without osteoid formation (Figure 7). Outside histology slides from the previously biopsied distal femur lytic lesion confirmed the initial distal femur brown tumor diagnosis (Figure 8). Subsequent positron emission tomography-computed tomography demonstrated no evidence for metastatic disease. An above-the-knee amputation was done (Figure 9), with histology confirming undifferentiated pleomorphic sarcoma (UPS) of the bone.

## Discussion

There has been concern for an association between PTH and osteosarcomas after in vivo and in vitro studies^11-13^ demonstrating a link in rat models and the current clinical use of PTH analogs as a treatment of osteoporosis. Although there have been a few case reports, a few retrospective human studies were not able to find an obvious association,^6,14,15^ with a need for further studies. Hyperparathyroidism in the pediatric population typically presents during adolescence, as with this case, and there appears to have higher incidence of bone involvement then adults.^16^ The effects of hyperparathyroidism on skeletally immature bone and development of sarcoma are not known, but an in vitro study^13^ suggests that exposure to PTH in younger rats may have a higher risk for sarcoma development than in rats exposed later in life. To our knowledge, there are no case reports of hyperparathyroidism associated with an UPS of the bone. Furthermore, no case has been reported of an UPS of the bone from a biopsy-proven brown tumor; however, UPS-like tumors may clearly arise in the setting of dedifferentiation or progression of other mesenchymal tumors (e.g. dedifferentiated chondrosarcoma) [17], suggesting that this patient's sarcoma may represent a very rare instance of progression of the brown tumor.

This case highlights an atypical presentation of undifferentiated pleomorphic sarcoma of the bone that developed in a young man with hyperparathyroidism at the site of a biopsy-proven brown tumor. The association of sarcomas with hyperparathyroidism is extremely rare with this representing the 10^th^ case in the English literature to our knowledge (Table 1).^1,2,3,4,5,6^ Of the now 10 reported cases, the age range is 15 to 69, with only 2 cases reported in men. No correlation can be made between the time of hyperparathyroidism diagnosis, treatment, and sarcoma location. Reported sarcoma pathologies include osteosarcoma, chondrosarcoma, and this case is the first report on an UPS. From these 10 cases, only 4 have either a corresponding pathologic diagnosis of a brown tumor or osteitis fibrosa cystica; 2 cases have radiographic evidence of other skeletal brown tumors, which the authors of this study believe is also significant because this is evidence of osseous changes related to hyperparathyroidism. In the four cases without these supporting findings of bone-related hyperparathyroidism changes, it is particularly uncertain whether the two cases reported by Keen et al are truly related to hyperparathyroidism, given that the chondrosarcoma diagnoses occurred in the background of other cartilaginous neoplasm; a well-established association is known between osteochondroma and enchondroma malignant transformation into chondrosarcomas. In our case, a potential confounder is the adjacent intramedullary nail. There is a rare reported association between sarcomas and orthopaedic prostheses/implants.^18-20^ Various bone and soft-tissue sarcoma subtypes have been reported with orthopaedic implants of differing metallic composition.^21^ The histological relation between these sarcomas and implants are not well delineated but initially favored to represent a foreign-body response^18^ leading to a hyperimmune state.^22^ In the review of orthopaedic implant-related sarcomas by Keel et al,^21^ the time range between implant placement and sarcoma diagnosis was 2.5 to 33 years (mean 11 years) in the 12 reviewed cases; our patient's intramedullary femoral nail was placed 2 year before sarcoma diagnosis. In our case, the UPS development appears adjacent to both the nail and brown tumor near the intercondylar eminence with pathologic fracture through the lateral femoral condyle. A limitation of this case is that it is difficult to delineate where the epicenter of the sarcoma is in relation because of extensive destructive changes that occurred by the time cross-sectional imaging was done.

**Table 1:** List of Other Reported Cases of Sarcomas Occurring in the Setting of Hyperparathyroidism

Case	Patient demographics	Time between hyperparathyroidism diagnosis and sarcoma diagnosis	Treatment of hyperparathyroidism	Sarcoma pathology	Sarcoma location
1 (Spiro et al)	46-year-old woman	3 years	Parathyroidectomy	Dedifferentiated chondrosarcoma^a^	Distal left tibia
2 (Spiro et al)	34-year-old woman	2 months	Parathyroidectomy	High-grade osteosarcoma	Mandible
3 (Mercuri et al)	56-year-old woman	Simultaneous diagnosis	Parathyroidectomy	High-grade osteosarcoma^b^	Proximal left tibia
4 (Vassilopoulou et al & Gagel et al)	69-year-old woman	Not reported	Not reported	High-grade fibroblastic osteosarcoma^a^	Right proximal femur
5 (Harell et al)	50-year-old woman	3 years	Untreated	Osteosarcoma	Mandible
6 (Keen et al)	66-year-old woman	Simultaneous diagnosis	Not reported	Low-grade chondrosarcoma arising from osteochondroma	Right scapula
7 (Keen et al)	36-year-old woman	Simultaneous diagnosis	Not reported	Low-grade chondrosarcoma arising from enchondroma	Left tibia
8 (Gagel et al)	15-year-old man	Simultaneous diagnosis	Not reported	Fibroblastic osteosarcoma^a^	Right femur and pelvis
9 (Gagel et al)	65-year-old woman	3 years	Not reported	Fibroblastic osteosarcoma^b^	Distal right femur
10 (our case)	22-year-old man	3 years	Parathyroidectomy	Undifferentiated pleomorphic sarcoma^a^	Distal right femur

a Brown tumor or osteitis fibrosa cystica on pathology

b Brown tumors on radiographs

This case demonstrates a young man with a history of adolescent hyperparathyroidism who ultimately developed an undifferentiated pleomorphic sarcoma of the bone in the distal femur. The difficulty in the diagnosis included osseous changes of hyperparathyroidism including multiple brown tumors, metal implant, pathologic fracture, and delayed presentation. This case highlights the possible rare association of hyperparathyroidism and sarcomas and difficulty in diagnosis because of the challenging clinical scenarios. Early recognition in the subset of patients is imperative by the clinicians and radiologist to improve clinical outcomes.
